# Immigrants Psychopathology: Emerging Phenomena and Adaptation of Mental Health Care Setting by Native Language

**DOI:** 10.2174/1745017901814010312

**Published:** 2018-10-30

**Authors:** Isabella Giammusso, Filippo Casadei, Nicolay Catania, Elena Foddai, Maria Chiara Monti, Giorgia Savoja, Crispino Tosto

**Affiliations:** 1 Department of Psychology, Educational Science and Human Movement, University of Palermo, Palermo, Italy; 2 Associazione PLP, Psicologi Liberi Professionisti, *Via* Pasteur, 65, 00144, Rome, Italy; 3 Istituto per le Tecnologie Didattiche, Consiglio Nazionale delle Ricerche, Palermo, Italy

**Keywords:** Immigrants, Mental disorder, Mental health service, Native Language, Psychopathology, Risk factors

## Abstract

Mental health of immigrants is an important social and clinical issue. Immigrants may report higher rates of mental disorders and lower levels of use of mental health service with respect to natives. The aim of the present work is to review recent findings of the psychopathology of immigrants and analyze how to adapt the mental care settings through the use of mother tongues. We searched the literature to individuate and review the most recent scientific articles focused on the psychopathology of immigrants realized in Europe. Moreover, we summarized the guidelines about immigrants mental health care and we focused on the barriers caused by language. We individuated 15 papers reporting data about mental disorders among immigrants and the related risk and protective factors. The articles reported information about psychosis, depression, anxiety, post-traumatic stress disorder, somatization and suicide rates. Risk and protective factors are individuated mainly among social factors (*e.g.* ethnic density effect, hosting countries' policies). Furthermore, immigrants encounter language barriers in the use of mental care services. The realization of cross-cultural training and the development of a working alliance between clinicians and interpreters resulted to be effective solutions even if these interventions are not frequently implemented. The extent of migratory flows and the related difficulties experienced by immigrants require attention and well-informed interventions. The high rates of incidence of mental disorder and the strict number of services who implement interventions taking into accounts fundamental aspect as language show that there is still a lot to do.

## 1 INTRODUCTION

The project ”Soluzioni Innovative per la Vulnerabilità E il Reinserimento sociale dei migranti” (SILVER), supported by the grant “FAMI - Fondo Asilo, Migrazione e Integrazione 2014-2020”, aimed at favoring the social integration of immigrants in the Sicilian territory. One of the outputs of the project is the realization of a study focused on emerging phenomena in the psychopathology of migrations and the modifications of the therapeutic setting and processes including native languages.

On 1st January 2017, 4.2% of the population of the European Union member state were non-member country citizens [1]. The interest on the mental health of immigrants is high since some studies showed that they are at high risk of mental illness [2, 3], even if differences related to motivation to migration, legal status, and cultural issues should be taken into account [4]. The risk may be incremented by many other variables. For example, pre-migration traumatic events and post-migration stressful experiences are factors that deserve to be carefully considered [5, 3]. This is particularly true for refugees, who experience a wide range of traumatic events before, during and after the migration journey and require a therapeutic approach focused on contextual factors and trauma-related symptomatology [6]. Moreover, the difficulty to reach the desired country and being reunified with surviving relatives has a negative impact on the success of clinical treatments among asylum seekers [7].

Moreover, immigrants show lower rates of use of mental health care services than natives because of a different kind of barriers [8]. These factors, combined, suggest that there are several new challenges in providing mental health care and there is still much to be done to correctly respond to immigrants necessities, especially in public health care. Thus, the present study aims to review the most recent findings on psychopathology among immigrants in Europe and the related risk and protective factors. Furthermore, an analysis of the most recent guidelines for mental health care for immigrants is provided, in which it is paid attention to the difficulties due to language barriers and how to deal with them.

## 2 EMERGING PHENOMENA IN IMMIGRANTS PSYCHOPATHOLOGY

We conducted a search of literature through the database Science Direct using the terms “psychopathology” and “mental health” combined with “immigrants” and “immigration”. We selected scientific articles published in international journals between January 2017 and June 2018 and focused on data gathered in European countries. We were interested in articles focused on the psychopathology of adult immigrants and the related risk and protective factors.

We found a total of 15 articles (Table **1**). Two of these studies were realized in Denmark [9, 10], 1 in Finland [11], 1 in France [12], 4 in Germany and Netherland [13-16], 1in Luxembourg [17], 2 in Norway [18, 19], 3 in Switzerland [20-22], and 1 was based on data gathered in 17 European countries [23]. They focused on psychosis [10, 11, 15, 16], depression, anxiety and Post Traumatic Stress Disorder (PTSD) [12, 17, 20-23], somatization [13, 14], and suicide rates [18, 19] and risk factors. The study realized in Finland [11] take into account all the psychiatric diagnoses coded by the International Classification of Diseases 10^th^ edition (ICD-10) [24]. Results show that immigrants report a lower incidence of mental disorders compared to natives, a counter-tendency result compared to other studies [2, 9, 10, 17]. Authors suggest the this is due to country policy and methodological factors. Finland has strict laws for immigration and they apply a tolerant and multi-cultural policy to those who enter the country. On the other hand, the design of the study does not let to collect information about the mental health of immigrants who do not access public health services.

### 2.1 Psychosis

First and second generation immigrants show high risk to report psychotic disorders and this risk appears to be related to socio-contextual factors in hosting countries [40]. Two of the most recent research in Europe take into account the ethnic density effect and urbanicity effect on psychosis rates among immigrants [9, 10]. The ethnic density effect is the association between the proportion of people of the same ethnicity in a community and the incidence of psychosis. High ethnic density can be a protective factor because of perceived social support, while low ethnic density is related to a higher incidence of psychotic disorders [41, 42]. Differently, urbanicity is a risk factor for psychosis [43]. First of all, rates of psychosis results to be differently distributed among subgroups [10, 11]. Specifically, immigrants coming from Africa showed higher rates of psychosis compared to other subgroups [10]. On the other hand, first-generation immigrants showed higher psychosis incidence compared to second-generation immigrants [9]. In both cases, immigrants resulted to be at higher risk compared to natives.

However, social environment conditions appear to be associated with psychosis rates. Previous studies [44] did not find evidence for the presence of urbanicity effect among immigrants and researchers hypothesized that urbanicity and ethnic density may interact. Even if the research objectives and samples are partially different, in general, ethnic density appears to be a protective factor against psychosis rates among immigrants [9, 10] and especially for the second generation of immigrants [10]. Moreover, the effect of ethnic density masked the effect of urbanicity on psychosis rates [9]. Thus, urbanicity may represent a risk which effect can be reduced by the support of their community. However, it must be noticed that risk factors for psychosis can be different between subgroups [10]. First generation immigrants have to face traumatic experiences in the country of origin and in relation to the journey and the process of migration while second-generation immigrants can be affected by socio-cultural barriers and isolation. Thus, the ethnic density can have different effects on different groups of immigrants. These results are relevant because these two studies are the first attempts to analyze this effect with large samples, by making comparisons between first and second generation immigrants and country of origin, and using a prospective population-based cohort study design. On the other hand, it must be noticed that both studies [10, 11] took place in Denmark thus results may be influenced by local factors (*e.g.* policies).

A study realized in Germany [16] focused on differences in social, clinical and criminological variables between immigrants from diverse countries who were referred to forensic psychiatric treatment. The major part of the immigrants from the Balkans, North Africa and the Middle East, and Sub-Saharan Africa suffered from psychotic disorders, while part of immigrants from southern Europe suffered from personality disorders or paraphilia. Results showed differences in the social characteristics of immigrants from Eastern Europe who reported the highest educational level. It appears that such rates of psychotic disorders are associated to the social defeat hypothesis [45] that implies that prolonged experience of adverse social and living conditions, interacting with biological factors, may increase the risk to develop a psychotic disorder.

Another study realized in Netherland aimed at analyzing differences in symptom expressions, cognitive and psychosocial functioning between natives and first and second generation immigrants presenting a First Episode Psychosis (FEP) and the outcome at 12 months follow-up [15]. This is probably the first attempt to study the entire range of FEP symptoms expression and the related outcomes and to compare them between natives and immigrants. First generation immigrants reported the higher deficit in neurobiological and social cognition performance compared to others at baseline, while natives showed lower levels of negative symptoms compared to others at follow-up. Moreover, all the subgroups showed improvement in symptoms remission, except for negative ones. the main predictors of functional and symptomatic outcomes were psychosocial functioning and annual income together with other baseline characteristics and symptoms manifestation while being an immigrant was not among them.

### 2.2 Depression, Anxiety and Post Traumatic Stress Disorder

First-generation immigrants report higher rates of depression compared to natives in Europe [46]. A study in Luxembourg [17] confirmed these results and showed that social support can act as a protective factor. Specifically, second-generation immigrants have the highest probability to develop depression, followed by first-generation immigrants and non-immigrants. Researchers suggest that these differences may be due to difficulties related to the integration of two different cultures that second-generation immigrants could face. In fact, difficulties in integration and perceived discrimination are risk factors related to depression [46].

Occupational social capital, the structure of contacts held by people in relation to different occupations, is another factor associated with depressive symptoms [21]. A large network of occupational contacts enhances the opportunities to obtain high-status occupations [47] and this is related to better mental health outcomes [48, 49]. The reviewed study [21] took place in Switzerland and focused on persons with Swedish or foreign (Iran and former Yugoslavia) born parents. First of all, people with Iranian-born parents reported higher levels of depressive symptoms compared to others and the occupational social capital partially explain the ethnic variation in depressive symptoms. People with an Iranian background showed notable variations in the prevalence of depressive symptoms by occupational social capital. Extensive access to manual occupations disclosed a marked increase in depression in women of Iranian descent. Status concerns and aspirations for high-status occupations appear to explain the increased prevalence of depressive symptoms in subjects with an Iranian background. Thus, perceived ethnic identity and subsequent career ambitions and prospects, rather than ethnic characteristics alone, appear to determine the propensity for depression in young adults of Iranian descent. Access to prestigious occupational contacts is proposed to serve as a buffering mechanism that may reduce the ethnic differences in depression.

Pre-migration and post-migration conditions can represent risk and protective factors. The association between post-migration stress and depression is confirmed in a study realized in Sweden in which the authors found also evidence that post-migration stress together with traumatic life events is associated with PTSD [20]. One of the reviewed study focused on immigration path and transnational ties as risk factors for mental health among sub-Saharan immigrants in France, with some differences due to gender [12]. The characteristics of migration paths covered pre-migration threatening experiences and the legal status in the hosting country and authors considered them as factors that can affect immigrant mental health. Similarly, international ties can have an effect on mental health. On the one hand, separation from the family and the moral obligation to take care of family members remained in the country of origin can be a stressor. On the other hand, perceiving support from the family can be a protective factor. Within the context of the characteristics of immigration paths, results [12] show that women’s mental health is affected by the experience of threat in the country of origin while men’s mental health is affected by having an illegal status in the hosting country. Conversely, the separation from a child was not associated with mental health, while having support from family and friend appears to be a protective factor in both men and women [12]. This information contributes to a deeper comprehension of the impact that certain political decisions and the social context can have on immigrants mental health. In fact, policy models influence depression rates among immigrants. Immigrants in a country with exclusionist policies show higher rates of depression compared to non- immigrants and compared to the country with inclusive and assimilationist policies [23].

Finally, we individuated a study focused on Post Traumatic Stress (PTS), depression and explosive anger symptoms among torture survivors [22]. The study showed that perceived controllability and emotion of anger and fear experienced during torture are associated with a more severe symptomatology among a group of refugees and asylum seekers from Turkey and Iran. Authors showed that fear mediates the relation between PTS and depressive symptoms and perceived controllability of the event, while anger mediates the relation between explosive anger symptoms and controllability of torture. Thus, emotions and the perception of lack of control experienced during torture are important factors on which the attention of mental health professionals should focus.

### 2.3 Somatization Disorders

Studies focused on somatization and symptoms presentation represent an important contribution to the understanding of psychopathology among immigrants. Immigrants from Turkey [13] and Vietnam [14] show symptoms presentation that differ from those of Germans and the variations can be due to differences in cultural expression of mental disorders. Turkish and Vietnamese immigrants reported higher levels of somatization compared to Germans [13, 14]. Women and first-generation immigrants from Turkey reported the highest level of somatization and those with the highest level of somatization also showed high levels of depression [13]. On the other hand, Vietnamese immigrants reported higher physical symptoms and panic-related symptoms while there were no differences in depression [14]. This symptoms presentation is in line with the cultural manifestation of psychopathology [50].

The differences in somatization rates between Turkey immigrants and Germans can be due to different factors. Turkey immigrants faced migration and acculturation processes that are stressful and they live worse socio-economic condition [51]. Moreover, Turkey immigrants could fear the stigma of mental illness and tend to express their emotional difficulties through somatization [52]. The information resulting from these studies are important because they contribute to understand these symptom presentations and consent to approach properly patients difficulties.

### 2.4 Suicide Risk and Suicide Rates

Research on suicide risk among immigrants has not yielded conclusive results. Some studies suggest that immigrants are at greater risk than natives [53], while other studies suggest the opposite [54]. We individuated two recent studies focused on suicide risk among immigrant compared to natives [18, 19].

First and second generation immigrants are at lower risk of suicide compared to Norwegians [18]. However, the authors analyzed suicide rates in detail and they noticed that Norwegian-born people with a foreign-born parent and foreign-born people with one Norwegian-born parent were at higher risk of suicide compared to Norwegian. Specifically, the latter were at higher risk of suicide compared to Norwegian if the country of origin were Asia or Central or South America.

The lower risk for suicide among first and second generation immigrants compared to natives falls inside the phenomenon defined “healthy immigrant effect” [55]. In some cases, immigrants show better health compared to natives. In the case of the research realized in Norway [18], it is hypothesized that these results are due to the fact that first generation immigrants are those with better mental health in their country of origin or that they experience an improvement in the quality of life once they arrive in the hosting country.

There are some social risk factors that have an impact on suicide rates among immigrants in Europe. Socio-economic variables as marital status, educational level, annual income and place of residence (in the capital area or not) have a different influence on immigrants and natives in Norway [19]. For example, being single contributes to raising the risk for suicide more among Norwegian than among immigrants while being married lowers the risk for suicide among foreign-born with a Norwegian-born parent. A low educational level is a risk factor for all the subgroups involved in the study except for second-generation immigrants. Low annual income is related to higher risk for suicide among all the subgroups with first-generation immigrants being the least affected subgroup. Finally, living in the capital area contributes to raise the risk for suicide among Norwegian and to lower it among first-generation immigrants.

Authors explain the fact that being single is not a risk factor for immigrants because the major part of them are young and not being married is not a matter of concern. Differently, being married can act as a protective factor for foreign-born with a Norwegian born parent because it can imply greater social support and integration. The educational level is related to skills and competencies and the possibilities to find a job, thus this variable affects immigrants and non-immigrants in a similar way. First generation immigrants are those less affected by low annual income. This could be due to the fact that, compared to the country of origin, their financial situation has improved in the hosting country. Finally, living in the capital area can be a protective factor for immigrants because they can have a higher possibility to find social support from other immigrants.

## 3 ADAPTATION OF MENTAL HEALTH CARE SETTING BY LANGUAGE

As reported in a recent review addressing studies mainly conducted in European countries [8], immigrants tend to show a lower degree of health care utilization than native populations. Specifically, despite the relevant proneness to mental health problems associated with migration and adaptation to the host country, the study revealed migrants' lower use of mental health services and only occasional higher use of emergency care due to several types of barriers that hinder the access to health services. Across European countries, migrants' tendency to poorly refer to mental health service has been recently confirmed by the results of a Norwegian study showing that specialist mental health care in Norway is still underused by a large proportion of the immigrant population, especially in the case of youth, and labor immigrants [56]. These findings are easily understood in light of a number of well-recognized barriers that hinder the access and adherence to healthcare provision [57-59]. With specific regard to mental health care, a lack of or poor mastery of the host country language, culturally determined beliefs on mental illness, and different expectations toward health care professionals may interfere even more with the diagnostic process and provision of treatment [60]. Taking into careful account immigrants’ special needs, the European Psychiatric Association (EPA) has already pointed out several basic requirements for mental health services which include, among others, the provision of culturally sensitive care and interpreting in mainstream or separate services [61, 62]. An additional EPA guidance [63] has been developed in order to explicitly state the need for a mandatory cultural competency training defined as a tailored response to immigrants' mental health care needs requiring mandatory knowledge, skills, and attitudes to increase outcomes of psychiatric care. Moreover, interpreters' availability and their use within a well-defined theoretical framework may be crucial in order to ensure quality service by reducing the risk for inaccurate diagnoses, non-engagement or early drop-out, and patients' dissatisfaction [61].

With specific regard to the use of interpreting services, the work by Bauer and Alegria in 2010 [64] reviewed empirical studies (also including European experiences) focusing on the evaluation of the consequences of host country language proficiency among patients and availability of interpreting services on psychiatric diagnosis, treatment provision, and on quality of patient-provider interaction. Overall, the use of professional interpreters may improve patients' disposition to self-disclosure and referral to psychiatric service while enhancing their satisfaction with care. Until then, however, only a few empirical studies had addressed the effect of levels of host country language knowledge and interpreting services on the quality of psychiatric care. It should also be considered that the availability and use of different types of interpreting services (direct, telephone-mediated, and mixed) across European countries resulted to be generally low in primary and mental health care services, with relevant differences between countries in the availability and use of the different types of interpreting services [65].

However, recent efforts have been done in the direction of the empirical assessment of the use of interpreters and cross-cultural training among European mental health services. For example, the work by Leanza *et al.* in 2015 [66] provides a qualitative analysis of interpreters and clinicians’ perspectives on factors involved in the development of the working alliance between interpreters and clinicians, and the definition of interpreters’ purposes and functions in two French and Canadian clinics. Even if the study originally aimed to evaluate the integration of interpreters in mental health care specifically dedicated to minors with an immigrant background, the Authors generalized their findings to overall mental health care provision. In interpreters and clinicians’ experiences, effective collaboration between interpreters and clinicians implied trust, respect, and mutual recognition of each one’s role. Training for both interpreters and clinicians, a longer service experience together with a well-defined theoretical framework helped stakeholders to recognize and appreciate the multifaceted role of interpreters as cultural informants, mediators, and even co-therapists. Interpreters and clinicians also appraised the positive effect of interpreting the therapeutic process and patients’ satisfaction with the treatment. Another study [67] evaluated the effect of cross-cultural psychiatric training on mental health care providers and refugee reception in Sweden. After the training, clinicians reported a relevant reduction in the negative consequences of lack of knowledge with regard to the asylum-seeking process and several health-related issues on their clinical experience. Moreover, participants reported an enhanced ability to empathize with the experience of refugees suffering from mental distress disregarding the relevance of communication barriers. On the other hand, cross-cultural training exerted no effect on poor collaboration and structural barriers involving the interaction between the different health organizations. The results from both these studies are noteworthy when considering that language barriers have been reported to be a major concern among clinicians in European primary care and mental health [68].

Finally, contributions from several European countries in systematically adapting their models of mental health care provision to immigrant population have to be mentioned. German, Norwegian, and Swedish mental health care models have been developing taking into consideration the provision of culturally and linguistically specialized intervention programs, including staff members with an immigration background, and providing additional cross-cultural training to health professionals [69]. Moreover, the work by Sturm *et al.* in 2017 [70] describes the implementation of a French culturally-sensitive service for child and adolescent mental health care. The presented Intercultural Consultation Service (ICS) mainly provides short-term therapeutic interventions to children and families with migration background. The service also aims to increase mental health professionals’ intercultural competencies through shared team discussions of individual cases.

Despite the recognition of services limitations in the above-mentioned experiences, such models of mental care provision represent noteworthy efforts to ensure high-quality clinical interventions that take into strong consideration immigrants specific needs for mental care. On the other hand, other European countries seem to suffer from the lack of tailored funding; for example, in the Italian health care system, interpreters are generally lacking in clinical settings and clinicians mainly use interpretation services provided by phone, with the exception of few clinics specifically built to serve some segments of immigrant population [71].

## CONCLUSION

The mental health conditions of immigrants deserve well-informed attention given the extent of migration flows and the fact that immigrants report lower rates of mental health services utilization compared to natives. The most recent information about the mental health of immigrants shows that they report higher rates of depression and somatization disorder compared to natives, while results are not univocal about suicide risk. In fact, it appears that in some cases, immigrants are at lower risk than native people. Moreover, many studies focused on risk factors related to mental health among immigrants, and especially on social ones. Ethnic density, for example, can be a protective factor against psychosis. However, it must be noticed that most of this information comes from studies realized in North Europe and reflects the mental health status of immigrants coming from a strict number of countries. Thus, results can not be extended to the entire immigrant population but they have to be read by taking into account these limitations. It should also be noticed that the expression of psychopathology is related to factors as immigrant status (*e.g.* asylum seekers, refugees), motivation to migration, and traumatic events.

One of the barriers encountered by immigrants is represented by language. Moreover, the nosographic categories used in the reviewed studies are those of the occidental cultures and this may limit the comprehension of immigrants disorders and the intervention efficacy. The enrichment of healthcare settings with interpreters and mediators have a positive impact on the efficacy of the service provided. Unfortunately, mediation services are often offered as external services managed by private organizations, which therefore are not fully integrated into the public service network. Policy makers and mental health professionals have to take into account these aspects when approaching immigrants needs.

## Figures and Tables

**Table 1:** List of the reviewed studies.

Author(s)	Sample/cases and data source	Mental Disturb(s)	Country where the study took place	Measure(s)/Codification
Bulla* et al.*, 2018	1419 immigrants referred to forensic psychiatric treatment in a hospital	Psychotic disorders, personality disorders and paraphilia	Germany	ICD -10 [24]
Dreher *et al.*, 2017	110 first generation Vietnamese and 109 German patients of two psychiatric clinics	Somatic symptoms and depression	Germany	Patient Health Questionnaire (PHQ) [25] subscales
Le *et al.*, 2018	108 refugees and asylum seekers from Turkey and Iran who were patients of two psychiatric clinics	PTS, depression and anger symptoms	Switzerland	Posttraumatic Diagnostic Scale [26]; Subscale for depression from Hopkins Symptom Checklist-25 item version [27]; item created ad hoc for the study.
Malmusi *et al.*, 2017	28333 non immigrants and 2041 persons born in a not “advanced economy” country individuated through data collected through the European Social Survey	Depressive symptoms	Cross-national	Center for Epidemiologic Studies Depression Scale (8 items version) [28]
Markkula *et al.*, 2017	184806 immigrants living in Finland and 185184 natives individuated through Finnish Central Population Register	All the psychiatric diagnoses according to ICD-10	Finland	ICD-10 (codes F00–F99) [24]
Miething *et al.*, 2017	5695 of Swedish citizens with at least one Iran-, former Yugoslavia-, and Swedish- born parent.	Depression	Switzerland	2 item from the Patient Health Questionnaire-2 [29]
Morawa *et al*., 2017	335 first and second generation immigrants from Turkey	Somatization and depression	Germany	PHQ-15 [30]
Pannetier *et al*., 2017	2468 immigrants from sub-Saharan countries visiting health care facilities	Anxiety and depressive symptoms	France	PHQ-4 [31]
Puzo *et al*., 2017	23 073 suicide cases occurred between 1969 and 2012 retrieved from National registers	-	Norway	ICD-8, for the years 1969 – 1985 (codes E950 – E959) [32]; ICD-9, for the years 1986 – 1995 (codes E950 – E959) [33]; and ICD-10, for the years 1996 –2012 (codes X60 – X84, Y870) [24]
Puzo *et al*., 2018	11,409 suicide cases occurred between 1992 and 2012 retrieved from National registers	-	Norway	ICD 9 for the years 1992 – 1995 (codes E950 - E959) [33] and ICD-10 for the years 1996 – 2012 (codes X60 - X84, Y870) [24].
Ruiz-Castell *et al*., 2017	1499 first and second generation immigrants and non-immigrants	Depression	Luxemburg	PHQ-9 [34]
Schoefield *et al*., 2017	2,195,684 persons living in Denmark individuated through the Danish Civil Registration System dataset. Data were merged with those retrieved from the Danish Psychiatric Central Register	Non-affective psychosis	Norway	ICD-10 (codes F20 - F29) [24] and their ICD-8 equivalents (ICD - 8 295.× 9, 296.89, 297.×9, 298.29 – 298.99, 299.04, 299.05, 299.09, 301.83) [32]
Schoefield *et al*., 2018	2,224,464 persons living in Denmark individuated through the Danish Civil Registration System dataset. Data were merged with those retrieved from the Danish Psychiatric Central Register	Non-affective psychosis	Norway	ICD -10 (codes F20 - F29) [24] and their ICD - 8 equivalents (ICD - 8 295.× 9, 296.89, 297.×9, 298.29 – 298.99, 299.04, 299.05, 299.09, 301.83) [32]
Steele *et al*., 2017	420 refugees and immigrants living in Sweden	PTSD	Switzerland	Harvard Trauma Questionnaire [35], Post-Migration Living Difficulties Scale [36], Cultural Lifestyle Questionnaire [37], and Hopkins Checklist-25 [38]
Stouten *et al*., 2017	46 Dutch, 60 first generation immigrant, and 56 second generation immigrant patients	Psychosis	Netherland	Positive and Negative Syndrome Scale [39] and a battery of measure for neurocognitive, social cognition, and psychosocial functioning assessment
